# How Canadian Law Shapes the Health Care Experiences of Women with Female Genital Mutilation/Cutting/Circumcision and Their Providers: A Disjuncture Between Expectation and Actuality

**DOI:** 10.1007/s10508-022-02349-w

**Published:** 2022-09-28

**Authors:** Danielle Jacobson, Daniel Grace, Janice Boddy, Gillian Einstein

**Affiliations:** 1 Dalla Lana School of Public Health, University of Toronto, 155 College Street (Room 500), Toronto, ON M5T 3M7 Canada; 2 Department of Anthropology, University of Toronto, Toronto, ON Canada; 3 Department of Psychology, University of Toronto, Toronto, ON Canada; 4 Department of Gender Studies, Linköping University, Linköping, Sweden

**Keywords:** Female genital cutting, Female genital mutilation, Health care inequity, Criminal code, Ethical debate

## Abstract

This study explored how the reproductive health care experiences of women with female genital mutilation/cutting/circumcision (FGC) were shaped. We used Institutional Ethnography, a sociological approach which allows for the study of social relations and the coordination of health care. From qualitatively interviewing eight women with FGC, we learned that they felt excluded within the Canadian health care system because they were unable to access reconstructive surgery, which was not covered by Ontario’s universal health coverage (Ontario Health Insurance Plan). We then talked with seven obstetricians/gynecologists (OB/GYNs) and learned that while it was legal to perform certain genital (e.g., female genital cosmetic surgery) and reproductive (e.g., elective caesarean section) surgeries commonly requested by Western-born women, it was not legal for them to perform other genital surgeries often requested by immigrant populations (e.g., reinfibulation), nor were these covered by OHIP (e.g., clitoral reconstructive surgery). From participants’ comparison of clitoral reconstructive surgery and reinfibulation to female genital cosmetic and gender confirming surgeries, it became clear that the law and policies within the health care system favored surgeries elected by Western adults over those wished for by women with FGC. We found that the law had an impact on the choices that OB/GYNs and the women they treated could make, shaping their respective experiences. This created ethical dilemmas for OB/GYNs and a sense of exclusion from the health care system for women with FGC.

## Introduction

Female genital mutilation/cutting/circumcision (FGC) has received increasing attention as women from practicing countries immigrate to the West and traverse health care systems (WHO, 2018). FGC refers to a diverse range of practices (types I to IV) that can include varying degrees of cutting and removal of portions of the external genitalia, as well as suturing the labia. Although there are a wide range of types of FGC, according to the World Health Organization typology, type III, which includes the cutting and suturing together of the labia minora (type IIIa) and/or majora (type IIIb; infibulation), is considered to be most severe in terms of the degree of cutting (WHO, 2018). In this article, we focus on type III FGC, and in particular, deinfibulation and other reconstructive procedures pertaining to this type of FGC.

Practiced across Africa, Asia, and the Middle East (WHO, 2018), reasons for carrying out FGC are also wide ranging and differ across diverse cultural communities. These varying reasons include, but are not limited to, association with purity and virginity (Johansen, 2017), marriageability (Abathun et al., 2016), and transition into being a woman (Boddy, 2016). In some countries where it is practiced, not having FGC may be considered as “a source of shame” (Koukoui et al., 2017, p. 8). However, contrary to popular belief surrounding male power and the carrying out of FGC, it is often the female family and community members who make the decision to practice FGC (Ahmadu, 2000; Einstein et al., 2018; Jacobson et al., 2018).

Laws against FGC formed in the West (Toubia, 1993) are suggested to have set the standard for the establishment of laws against FGC globally (Andro & Lesclingand, 2016). In practising countries, such laws have established the “perception of an international consensus to eradicate female genital cutting” (Boyle & Preves, 2000, p. 704). In our previous work, we found that the goal of eradicating FGC on a global level was entangled in anti-female genital mutilation (FGM) public conversations. Although those who engaged in/co-produced anti-FGM discourse (such as media and news outlets) often aimed to “protect” uncut girls and women and advocate for women with FGC, these discursive strategies also often resulted in the stigmatization of women, their bodies, and their cultures (Jacobson et al., 2021). This is not the first instance in which the “stigmatizing nature of much current activist, academic, and social-policy discourse” regarding FGC has been discussed (for example, see Earp, 2020a, p. 1).

In Canada, an amendment to the Criminal Code first made FGC illegal in 1997 based on the notion that it violates human-rights. It is important to note that there are laws criminalizing FGC in countries across the globe, which makes this issue a global one and not one specific to the Canadian context. However, we focus on Canadian law and context in this article given that it is the location where we conducted this research. The law states that “The Parliament of Canada has grave concerns regarding violence against women and children and, in particular, child prostitution, criminal harassment, and female genital mutilation” (Bill C-27, 1997, p. 4). The language of this law associates FGC with “harassment,” “mutilation,” “violence against women,” and “child prostitution.”

It is important to understand how this law and the normative standards it enforces are implicated in reproductive health care. This includes what adult women with FGC can choose for their bodies given that ideas within the law become “standardized” in “contemporary administration and governance” (Mykhalovskiy, 2008, p. 141). This means that the law can often become regulated and normalized through administrative and governing bodies. This includes both the federal and provincial governments, and professional societies (i.e., Society of Obstetricians and Gynecologists of Canada [SOGC], College of Physicians and Surgeons of Ontario [CPSO]) that translate such laws to professionals. While we do not imply that statements from such professional societies are driven solely by the law, this is one method through which laws can become communicated and translated to professionals.

Under this law (Bill C-27, 1997), reinfibulation, which involves the re-suturing together of the labia (i.e., after giving birth or in some cases to re-establish a sense of virginity; Berggren, Musa Ahmed, et al., 2006), is illegal and carrying it out is deemed as professional misconduct for doctors (Perron et al., 2013; WHO, 2010). However, deinfibulation, which involves an incision made to the scar where the labia were sewed together (exposing the urethra and vaginal opening; Nour et al., 2006; Safari, 2013), is legal. Women with FGC may undergo deinfibulation to treat vulvar pain (Ezebialu et al., 2017), when giving birth, or when their uterus, cervix, or vagina requires assessment or surgery (Johnsdotter & Essén, 2021). Even though reasons for performing deinfibulation are often medical, the deinfibulation procedure itself would also make the vulva resemble what is considered to be “normal” in the West. While deinfibulation, which may instantiate “normality” in the West, is legal, reinfibulation, which is considered to be a “mutilating” practice, is illegal. This may point to the influence of Western body norms on the formation of these laws.

What is and is not legal with regard to genital surgeries has sparked ethical debates surrounding what is and is not considered to be acceptable for medical or therapeutic reasons and what does and does not constitute an exception to the laws against bodily harm (Cook et al., 2002; O’Neill et al., 2020; Shahvisi & Earp, 2019). These debates include the opposing view that male circumcision, which is legal and involves the removal of the foreskin (Blank et al., 2012), is accepted, while FGC is not legal and is rejected (Darby, 2016; Earp, 2015, 2020b; Möller, 2020). We do not suggest that male circumcision and FGC procedures are equivalent, but rather highlight how from this deliberation comes advocacy for evaluating genital surgeries according to bodily autonomy and informed consent (Earp, 2015, 2020a; Svoboda, 2013; Townsend, 2019).

Complicating the debate are other elective genital surgeries aimed at “normalizing” or enhancing Western female bodies, including female genital cosmetic surgery (FGCS). FGCS can include “labiaplasty (labia minora reductions), labia majora ‘augmentations’ (tissue removal, fat injections), liposuction (mons pubis, labia majora), vaginal tightening (fat injections, surgical tightening), [and] clitoral hood reductions” (Boddy, 2016; Braun, 2005, p. 407). Despite the fact that for any given individual, FGC may also be carried out for many reasons relating to normalizing and beautifying the body (Einstein et al., 2018) and sexuality (Tiefer, 2008), FGCS are accepted and legal, while FGC is not (Boddy, 2016, 2020; Johnsdotter & Essén, 2010; Manderson, 2004).

Finally, although not a comparison to another surgery, there is an ethical debate regarding the efficacy of procedures deemed medically unnecessary, like female genital reconstructive surgery (such as clitoral reconstructive surgery; Sharif Mohamed et al., 2020). Reconstructive surgery, for women with FGC, involves bringing the remaining portion of the clitoris, which had been previously cut, into a “normal position” (Foldès et al., 2012, p. 134). This procedure is thought to, “…reduce clitoral pain, improve sexual pleasure, and restore a vulvar appearance similar to that of uncircumcised women” (Abdulcadir et al., 2015a, p. 275). This suggests that what is thought to be the “normal” clitoral position in the West is that of a woman without FGC.

These debates and comparisons are important in considering equity under the law, especially if the law relies on cultural standards to ensure “normative” bodies. The law not only dictates what adult women are and are not legally allowed to do with their bodies, but ultimately affects the possible stigmatization of nonconforming bodies, which may permeate into the health care encounter. It is not currently known how the law shapes women with FGC’s experiences with reproductive health care, nor how it organizes doctors’ work of caring for them. We explored this issue for women with FGC and their obstetrician/gynecologists (OB/GYNs) in Toronto, Canada.

## Method

We used Institutional Ethnography (IE), a social-justice-oriented approach created by Smith (1987, 2005, 2006), to carry out this work. We did not enter into this research asking questions about the law. Instead, we wanted to better understand the social relations that shaped women with FGC’s experiences of reproductive health care to reveal *how* these experiences come to happen (Campbell, 2003). Social relations are the connections between people and activities within an institution and are observed in order to understand how they are organized beyond any one account (Campbell & Gregor, 2002).

In this article, one social relation that we identified was the law. This is not the first IE study to explore the law and policy coordinating social relations surrounding issues of health. For example, laws have been studied to understand how they are “‘put together’ to respond to the social and political challenges of HIV and AIDS” (Grace, 2013, p. 590); particular public health policies surrounding alcohol use in England were found to inform how licensed businesses conducted their work (Grace et al., 2014); legislation was found to shape online sexual health services and access for gay, bisexual, queer, and other men who have sex with men in Ontario (MacKinnon et al., 2020a). While IE has been used to investigate how the law shapes everyday health experiences of groups marginalized by oppressive social structures, the application of this strategy to the reproductive health care experiences of women with FGC is novel.

We began this investigation by listening to women with FGC talk about their health care experiences and noticed a disjuncture in their accounts. Through a process of analysis, we linked up the disjuncture as told through their experience to the law. This linking involves beginning with embedding the study in the accounts of participants’ everyday experiences and looking up toward institutional processes which may regulate or shape those experiences. A disjuncture occurs “between different versions of reality” when knowledge from a ruling perspective (i.e., the law) differs from the experiential perspective (i.e., women with FGC’s experiences; Campbell & Gregor, 2002, p. 48). The disjuncture is therefore the analytic entry point into the research and is revealed through accounts of everyday people’s lives (Ng et al., 2016).

Thus, we began with women with FGC’s experiences and linked up to the material relations that shape them. Women with FGC described how they were welcomed into Canada, which is portrayed as a “multicultural” society, yet felt excluded without access to particular genital surgeries (i.e., reconstructive surgery) despite similar procedures being afforded to other citizens. We then pulled at this “thread” (DeVault & McCoy, 2006) by interviewing OB/GYNs to understand institutional factors shaping the reproductive health care encounter, including women with FGC’s described lack of access to this surgery. We continued by linking these experiences on the ground up to the law (i.e., Bill C-27 and related health care policy), which contributed to women with FGC feeling discriminated against in the reproductive health care system.

This study was approved by the University of Toronto Research Ethics Board. We recruited both women with FGC and OB/GYNs with the help of a community health center, through referrals, and by posting flyers on public flyer boards, at hospitals, and at the University of Toronto. The most fruitful method of recruitment was via referral. Women with FGC were told that we would like to better understand their health care experiences, that one-on-one interviews would be confidential, and that they would receive compensation of $40 CAD. OB/GYNs were told that we would like to better understand their experiences caring for women with FGC and that one-on-one interviews would be confidential. A community advisory group (CAG) including two women with type IIIb FGC in Toronto helped to guide this study, offering direction and feedback on our method, measures of sensitivity, approach to interviews, and if the study would be constructive for the community.

Fifteen one-on-one, qualitative interviews were conducted by the first author: eight women with type IIIb FGC, and seven OB/GYNs. In order to protect the privacy of all participants, we did not recruit patient–doctor dyads. Women with FGC were included in the study as long as they self-reported having had type IIIb, were over 18 years old, and accessed reproductive health care in Toronto and/or the greater Toronto area (GTA) within 10 years. All women with FGC were compensated with $40 CAD. Women with FGC were from Sudan, the Gambia, Somalia, and Kenya and had immigrated to Canada between 1993 and 2019. These participants had FGC in their natal country (the countries where women were born) between the ages of four and seven and ranged from 25 to 70 years of age at the time of interview. Their education ranged from high school to a master’s degree. Seven women had between one and four children; one had no children.

Doctors were included in the study as long as they were a certified OB/GYN, had worked in Toronto and/or the GTA, and had experience caring for women with type III FGC. OB/GYNs who participated in this study were born in Canada with the exception of two from countries overseas. (We have not specified which countries to protect doctors’ identities.) They ranged from 35 to 75 years of age at the time of interview and had practiced medicine from six to 40 years. OB/GYNs in this study had practiced medicine in Toronto or the GTA, had treated at least one woman with FGC in this region, and had received at least a portion of their medical training in Canada (and all but one in Ontario). All, except for one, identified as women. Further details about the larger study of which this article is a part can be found in a separate report (Jacobson et al., 2021).

We entered into the research by interviewing women with FGC to learn what they had to say about their reproductive health care experiences. We then interviewed OB/GYNs. Open-ended questions prompted participants to share the step-by-step details of their experiences, voicing their own concerns about their navigation of the reproductive health care system. Interviews took place where participants were most comfortable, including the University of Toronto, local hospitals, a community health center, and over the phone. All participants provided informed consent, completed a socio-demographic questionnaire, chose a pseudonym, and were informed of their right to stop participation at any point without any consequence. Each interview was between 1 and 2 h long. For two women with FGC who preferred to conduct the interview in a language other than English, a reputable interpreter whose services were previously utilized by the community health center was recruited and was briefed on anonymity, confidentiality, and the study’s purpose. Only English translations of the interviews conducted in another language were transcribed. All interviews were audio recorded, transcribed, and checked for accuracy by both a research assistant and the first author.

We have limited reporting the demographics of participants in order to protect their identities; the community of women with FGC as well as the OB/GYNs who are known to treat them is close-knit. During the interviews, we used the same language that women with FGC and doctors did when referring to FGC. For example, if women used the term “FGM,” we mirrored that language during the interview.

Data analysis was an iterative process that occurred throughout the research and through discussion with the other team members. Audio-recorded interviews and analysis of texts such as transcripts and guidelines from professional societies (i.e., SOGC) helped to reveal the social relations embedded in participants’ experiences (DeVault & McCoy, 2006). Analytical thinking began during interviews by checking and rechecking our comprehension of the described experiences and how they were shaped, either validating or correcting our understanding as we talked with more participants (Campbell & Gregor, 2002). This is an important part of data analysis in IE, since it is the participants who reveal how their experiences happen as they do.

We indexed transcripts as an analytical tool, which relied on grouping work within an institution (Rankin, 2017). For example, we grouped together descriptions of the work that OB/GYNs did to navigate ethical dilemmas that came up in their medical work. We used NVivo software, highlighting certain portions of text within each transcript and grouped these portions together within a single digital folder. Having all instances of particular kinds of work to which participants engaged grouped together organizationally helped us better understand the connections between people and texts with regard to these dilemmas (Rankin, 2017). We also continuously went back to the transcripts, reading them over multiple times to further reflect on the social relations that shaped women with FGC’s reproductive health care experiences (Smith, 2006). Each account added to a better comprehension of the experience as a whole beyond any individual account (Campbell & Gregor, 2002; DeVault & McCoy, 2006).

## Results

### The Inaccessibility of Reconstructive Surgery

Women with FGC in this study described how they wanted to be “normal” in Canada, but instead, felt “different.” While Lena said, “When I came here, I felt that I was different,” Fatima explained, “We don’t feel like the normal people…Canadian or everybody—they don’t cut.” Fatima described how deinfibulation was one means of making her vulva “normal” in Canada: “After that [deinfibulation] you feel you are normal…I am normal.” However, other women, like Lailatou, said that they wanted clitoral reconstructive surgery to restore their vulvar appearance and sexual function: “I want to regain back, I want to have my clitoris…I want to be able to have a healthy and enjoyable sexual life. I need that! It’s my right.”

Lailatou described working hard to find an OB/GYN in Canada who would perform clitoral reconstructive surgery. She turned to Google to find this: “Sometimes I just go looking at the reconstruction options because I am so much into that. So, I Google that and try to hear from people who do it mostly in the U.S.” However, she was not successful in finding an OB/GYN in Canada to perform the procedure: “In Canada, I don’t think we have much information about it, sadly.” Rama also described difficulty finding a doctor in Canada to perform clitoral reconstructive surgery: “Obviously I would want [reconstructive surgery] if I could [laughs]! But, I don’t know that it’s happening here [Canada].”

When a Canadian OB/GYN could not be found to perform clitoral reconstructive surgery, some participants turned to American OB/GYNs offering the procedure for free. Rama connected with one American OB/GYN over social media private messaging: “He [the American OB/GYN] told me about a centre he runs in [American city], and he said he runs free FGM reconstruction surgeries there.” However, Rama was unable to take advantage of this opportunity because this OB/GYN retired.

Even when participants were able to find a doctor in Canada to perform this surgery, financial considerations made it inaccessible. Rama said, “My doctor [asked], ‘Do you want me to talk to people who can maybe do corrective [reconstructive] surgery?’ …I was like, ‘Yeah I would love to if it’s free!’” Participants described how they did not have the financial means to pay for this surgery and could only have it if it was “free” or covered by the Ontario Health Insurance Plan (OHIP). Lailatou explained, “One problem that I have is in Canada for instance…it’s not covered by OHIP, the reconstruction. And it’s really expensive! …But the thing is, I cannot because I don’t have the money, the means to pay for it.” The lack of coverage for clitoral reconstructive surgery was framed here as a “problem,” as women with FGC in this study described how this barrier prohibited their ability to improve their sexual health, which they viewed as their right as Canadian residents.

Women in this study described immigrating to Canada with the impression that it was a “diverse” country. Lailatou said, “Canada is like a benevolent country. It is multicultural. You know, it is a dream heaven for so many people in other parts of the world.” However, we observed a disjuncture when she became aware that her lack of access to clitoral reconstructive surgery, both in her ability to find a doctor to perform it and the lack of insurance coverage, differed from the portrayal that all residents have access to the health care that they perceive they require in Ontario. Lailatou continued, “Canada takes people who are from so many immigrant practicing communities…[but] FGM is not seen as a strong Canadian problem, right? …Because, oh yeah, you know, it is illegal here…So it is not a Canadian problem.” She came to learn that FGC was often viewed as an issue that only affects immigrants and was therefore not dealt with successfully by the health care system, which did not cover the costs of their described needs (i.e., clitoral reconstructive surgery).

This lack of coverage made Lailatou feel that her reality was not taken into consideration. Part of her plea for OHIP to “cover many other realities” was her desire to “just be accepted” and to “erase that part of me” that she no longer saw as normal. To her, such coverage would signify belonging:

Canadians are accepting immigrants. And if you accept people, you accept their entire package…It is essential that we talk about these things. If we don’t, nobody would get to know. We would be crying silently in our bedrooms…It’s [Canada] a space that we are. Promised to freely live in, right?! So, if I am accepted, I want to be accepted as a whole, not just a part of me, bits and pieces, like the other part should be erased and whatever? It’s an integral part of FGM.

For women with FGC in this study, a part of Canada’s promise for them to “freely live in” what was supposed to be an inclusive country was bodily autonomy. However, as an adult with OHIP coverage but unable to have clitoral reconstructive surgery covered, Lailatou did not feel that she was *really* able to exercise this right:

[Women with FGC] should be able to have the ability to reverse their condition if they so wish to…This is where I feel the OHIP is definitely not helping…So that’s why opening up of OHIP to cover many other realities of the people coming in [to Canada], comes into play…With the fact that OHIP is not covering this…It’s [the practice] an integral part of me now. I cannot erase it. I cannot undo it. I cannot. It’s just part of me. So, what the government can do is just open up, to give us more options to be able to determine what. We want to do with our bodies.

What solidified her feeling of being frustrated, discriminated against, and unaccepted in Canadian society was knowing that other genital surgeries were covered for Canadian-born adults who did not have FGC. Lailatou particularly compared how gender confirming surgeries for trans individuals were covered by OHIP, but how clitoral reconstructive surgery for her was not. This example highlights that Lailatou picked up on how some individuals were afforded their right within the law to have “normal reproductive function or normal sexual appearance or function” (Bill C-27, p. 6), regardless of the difficulty of accessing procedures that would allow this appearance, but she was not afforded this same right:

Today, if I want to change, if I have a different sexuality or if I am more comfortable in a different sexuality, I have an option! There is an option [for trans individuals]. So, let this [reconstructive surgery] also be an option!

When Lailatou described individuals having a “different sexuality,” she was referring to individuals who do not identify with the gender traditionally associated with their genitalia. She highlights here that if an individual is a man but has a vulva, or is a woman but has a penis, they have the “option” to have their gender confirming surgery paid for by the government. Though Lailatou was the only person to make this comparison, Lailatou used this example to highlight an issue of inequity that she felt was unfair. This health care coverage inequity reflected the limitation within the law of the genital surgeries often requested by, but not financially accessible to, immigrant women with FGC. It is important to note that although not necessarily visible from participants’ standpoints, there are many hardships and barriers that trans individuals face when trying to access gender confirming surgery (MacKinnon et al., 2019; MacKinnon et al., 2020b).

Dr. Cudjoe confirmed that “you would have to charge the patient” to perform clitoral reconstructive surgery, if the doctor had the expertise to do so, and stated their view that the lack of coverage for reconstruction had to do with the discrimination that women with FGC face:

The interests of these people [women with FGC] by and large has not been served well by the health care system in this country. OHIP payments for instance do not recognize [clitoral reconstruction]...The only payment is for deinfibulation. That is the only thing they will pay for…It’s all part and parcel of the neglect that these people have experienced.

Not only did women with FGC in this study recognize the lack of coverage for clitoral reconstructive surgery, their doctors also recognized it as a form of implicit discrimination within the health care system.

### Linking up the Disjuncture Observed in Women’s Accounts with OB/GYNs’ Accounts of Ethical Dilemmas and the Law

The accounts of women with FGC in this study revealed that despite being welcomed into what was portrayed as a “multicultural” country, they felt excluded as they did not have access to particular genital surgeries despite similar procedures being afforded to other citizens. This disjuncture was further elucidated from OB/GYNs’ accounts in the present sample of the types of surgeries they could legally perform for women who do not have FGC. OB/GYNs in this study explained that the law presents inequities that deny certain types of health care to women with FGC, especially when not deemed medically necessary. This created ethical dilemmas for the OB/GYNs.

Though participants with FGC did not voice the wish for reinfibulation, OB/GYNs in this study did say that some of their patients with FGC requested reinfibulation. Dr. GK and Dr. Beatrix, OB/GYNs who had been asked by women for reinfibulation, thought deeply about the rules surrounding it. Dr. GK was specifically asked to perform reinfibulation of the labia minora. They discussed becoming informed about the rules surrounding such procedures from professional societies. In doing so, they noted that although in Canada it is illegal to reinfibulate, it is legal for women to choose more dangerous surgeries like elective C-section:

You know SOGC says that we can do elective C-section at term if the patient requests it, which is a lot more invasive [than reinfibulation of the labia minora]. We [call this] a woman’s right to choose. We give women a right to choose the C-section even though it’s got way, way, way more morbidity, but not for a woman to choose to be sewn back up…And then the next one walks in and wants an elective C-section and I say, ‘sure fine I’ll give you maybe internal adhesions and maybe pain and all sorts of things and maybe problems with your next birth, but sure I’ll do that!’ But then I’m not allowed to sew up some mucosa [labia minora]?

With the possible dangerous outcomes related to elective C-sections, or in other words the ratio of risks compared to benefits of this procedure when elected in non-emergency or medically unnecessary scenarios, OB/GYNs in this study thought it unfair that women could elect to this procedure but not the procedure to reinfibulate, which they did not see as carrying any greater risk than a C-section. The point was not to advocate for reinfibulation, but to highlight inequities in what adult women with FGC could decide they wanted for their bodies based on what was accepted as “normal” under the law. The law’s limiting of women’s bodily autonomy was further contradictory to the discursive framing of women’s “right to choose” within professional societies.

These legal inequities sparked ethical dilemmas for doctors in this study about how their own opinion on FGC influenced their medical work. Dr. GK said:

It was a total ethical dilemma because I respect a woman’s right to choose but I find it. [reinfibulation] abhorrent. But am I judging her based on her culture? Did I not respect. her right to choose [by not performing the reinfibulation]? I do believe that maybe by not. Sewing her up I did not do what was her choice.

Although Dr. GK neither supported elective C-sections nor reinfibulation, they wondered how their opinion of FGC as “abhorrent” and adherence to the law by not performing reinfibulation was tangled up in the prohibiting of women’s “right to choose.”

OB/GYNs in this study also questioned whether it was “ethical” to prohibit a woman from restoring her genitalia to the way they had been her entire life. Dr. Beatrix said:

There is the debate about adult women who, after giving birth, ask to have their stitching. redone [reinfibulation]. And this adult woman has known her body in a particular configuration all of her life, and the ethical guidance is you don’t do it. And we really struggle with that.

Regardless of their opinion on the procedure, the OB/GYNs we interviewed highlighted their struggle in questioning the “ethical guidance” within the law. If a woman had been infibulated her entire adult life, but upon giving birth in Canada became deinfibulated, was it ethical to not allow her to reinstate her genitals to what she considered as “normal?”.

The ethics of medical practices resulting from the Canadian law was not only questioned by comparing reinfibulation to elective C-section, but also by comparing it to FGCS, an elective procedure for women without FGC that is often not medically necessary:

There are adult women who are allowed to make choices around cosmetic surgery that. They undergo. And yet, we take a different lens to African women who are asking to have. A cosmetic appearance that is pleasing to them. Yes, there is an ethical guideline. Is it. Straight forward? No. Anything but. (Dr. Beatrix).

The “ethical guideline” put forth by the law clearly discriminated against women with FGC as it allowed Western women to “choose” cosmetic surgeries, but did not allow women with FGC the same right for similar procedures. Despite OB/GYNs’ view in the present sample of both cosmetic procedures as reconfiguration of the labia, they were legally and professionally bound to follow such rules.

Despite these dilemmas, OB/GYNs in this study were explicit that the solution was not necessarily to *allow* reinfibulation; they described the possible negative repercussions of condoning such a procedure. Dr. GK explained that if they did perform this procedure for adult women, that such women may expect the same for their daughters:

It’s a slippery slope because [what if] now she comes to me with her daughter and says. ‘you sewed me up after my thing [delivery], can you do the same procedure on my. daughter?’ I think, you know ethically…It’s do no harm. I think sort of Western women. have a lot of abhorrence about this…You know, we do get those ethical sorts of choices. And you don’t always make the right choice…You know, you sort of cop out a little bit.

Although OB/GYNs in this study believed in the law to some extent, they also believed that women were being denied ownership of their bodies. They questioned if they really were “doing no harm” by following the law or if they had “copped out a little bit” by not allowing women with FGC their “right to choose” the genital surgery they wanted for their bodies. When they considered the possible repercussions for what women with FGC might want for their children they imagined a “slippery slope,” which helped them see the law as supporting their ethics. It is important to note that women with FGC in this study indicated that if given the choice now, they would neither want FGC for themselves, nor for their daughters. Fatima said, “My kids—no. My daughter, we didn’t do that [the practice] for her.” Similarly, Asha explained, “My daughters were saved, they did not go through the cuts.” Thus, the assumption that there is a “slippery slope” may not be well-substantiated.

### The Law: Bill C-27

Participants with FGC in this study described a lack of inclusivity of OHIP policies which prohibited their access to clitoral reconstructive surgery. OB/GYNs interviewed described inequity in the law which denied their patients with FGC the ability to determine what was best for their own bodies in spite of other women being allowed to elect to Western reproductive surgeries considered more dangerous. Thus, the aforementioned disjuncture that Canada accepts women with FGC into the country but its health care system does not cover the costs of what such women feel they need is further linked up to the law. The law regulates a woman with FGC’s “right to choose” and thereby creates serious ethical dilemmas for OB/GYNs.

Women with FGC in this study knew about Canadian laws against FGC and related policies. However, they were unable to identify the specific sources of this knowledge. As Lailatou described: “It is illegal here [Canada] so nobody can cut their kids here.” Fatima demonstrated knowledge in her careful word choice for describing the repairs made to her vulva after giving birth in Canada: “You know, I was lucky because I found this doctor and she knew how to close this area again…Not closing! But she is fixing!” Distinguishing between the word “closing” (often associated with reinfibulation, which is illegal) and the word “fixing” (not usually associated with this illegal procedure) demonstrates Fatima’s knowledge that she and her doctor could get into legal trouble if the word “closing” was mistakenly interpreted as her having been reinfibulated. To ensure that her meaning was understood, Fatima explicitly explained the law as common knowledge to show her following of it: “If I go back to my [natal] country they have to sew more [reinfibulation]. But here it’s not legal to do that. We don’t do it here. Yeah, they don’t do that here.” OB/GYNs in this study also indicated that their patients with FGC were knowledgeable about what could and could not be legally done for them. Dr. GK explained, “I think that most–a lot of times they’re pretty savvy people. They know that there’s rules here in Canada, and they won’t be able to have something done that crosses our boundaries.”

Women with FGC we interviewed engaged more indirectly with what was written in the law on FGC by understanding how it was reflected in the types of medical procedures, like reconstructive surgery, that were not covered for them under OHIP. While the Criminal Code has the stated mandate to protect women’s “human-rights and fundamental freedoms” (Bill C-27, 1997, p. 1), it at the same time limits what they are and are not allowed to do with their bodies by categorizing particular genital procedures as “mutilation,” thereby making them illegal. Though genital procedures like clitoral reconstructive surgery are legal, OHIP’s lack of coverage for such procedures mirrored this legal inequity of adult women supposedly having but being restricted in their ability to engage in their rights and freedoms.

OB/GYNs in this study similarly demonstrated knowledge about Canadian laws against FGC. Dr. Fanny explained, “It’s clearly established, literally in the law…It’s a criminal offense in Canada to do this to somebody.” In particular, it was Bill C-27 (1997), the amendment to the Criminal Code of Canada, that first made FGC practices illegal, associating them with “violence,” “criminal harassment,” and “mutilation.” It is worth noting that in IE terms, the institutional language used in Bill C-27 works to reduce the complexity of the everyday world by standardizing particular ideas like rules against FGC (Smith, 2005).

One way that such legal ideas and language become standardized is through particular texts such as guidelines from professional societies. The OB/GYNs we talked with traced their source of legal knowledge to organizations that communicate such information to them like the SOGC, CPSO, and World Health Organization. Dr. Rose explained, “Guidelines are important, we follow them so that we’re up to date.” OB/GYNs described turning to these guidelines when they had questions, were unsure about treatment expectations, wanted new insights on treatment, or wanted more information about FGC and the law. Dr. GK explained that the SOGC was the governing body for OB/GYNs in Canada, which sets the standards on treating women with FGC ethically, morally, and legally:

In Canada, we tend to follow professional guidelines. We try to, anyway…You. internalize them. So, I know that SOGC does not support FGM/C. They do not allow us. to participate [in FGC] in any way, obviously…You would go to the [SOGC]. guidelines more than the government legal stuff…its confusing. It’s our professional. guidelines. It changes our [medical] practice.

Citing the criminal code in guidelines such as ones from the SOGC (Perron et al., 2013, 2020) is therefore thought to help doctors understand the legal rules surrounding FGC and their liability.

Dr. Beatrix also described that doctors look to such organizations for legal rules on FGC: “The SOGC has actually written a guideline that we follow, and the World Health Organization [also] has one. And those are that you do not perform female genital cutting, and you do not reinfibulate.” Another professional society that translates the law to doctors is the CPSO (CPSO, 2011). Dr. Fanny explained, “It’s established in our college guidelines…you’re not allowed to do that [FGC] to anybody…inclusive of if it [the infibulation] were to tear open during delivery…you’re allowed to make the edges cosmetic and hemostatic, but you’re not allowed to reinfibulate somebody.” Professional organizations were therefore important to standardizing legal information to the medical doctors working with women with FGC.

OB/GYNs in this study also knew that there were consequences if they did not follow the legal and professional guidelines. Dr. Beatrix explained, “Where there is a law around it, then you’re liable to criminal prosecution if you deviate from the guidelines.” The fear of being prosecuted put further pressure on doctors to make the legal choice when facing ethical dilemmas, even when they viewed it as inequitable.

The ideas and language within the law, translated to doctors via professional societies, are also oftentimes stigmatizing (i.e., associating FGC with “criminal harassment”) and normalizing (i.e., indicating that women are entitled to “normal” vulvar appearance and function). For example, Bill C-27 (1997) further states that surgery to the vulva is only acceptable for the purpose of restoring “normal reproductive…[and] sexual appearance or function” (p. 6). Importantly, there is no definition of what “normal appearance” consists. For example, OB/GYNs in this study explained that what was not considered to be “normal” was the re-suturing together of the labia (reinfibulation)—a procedure that was normal in the countries from which women immigrated. Dr. Janice explained, “You can’t close them back up [reinfibulation]. You can’t interfere with what’s there…You would try to give them a more normal appearance…You would try to sort of reproduce normal anatomy.” The notion that “you can’t interfere with what’s there” once a woman has been deinfibulated indicates that doing so would deviate from what is legal and thus “normal.”

OB/GYNs in this study described that they could only deinfibulate women so that their labia were no longer stitched together to more closely resemble a “normal” un-stitched vulva, consistent with Western practices and the law. Therefore, inherent in the legislation is the implicit assumption that “normal” vulvar appearance is based on bodies (that have not undergone FGC) in the Western context in which this document was made. The law thus coordinates the conceptual knowledge of what is “normal” through dictating which procedures women can access.

Therefore, Bill C-27 guarantees women their rights and freedoms but also limits what adult women with vulvas not conforming to the Western norm can do with their bodies. The exclusionary aspects of the law become foisted on doctors through professional guidelines and on women with FGC, through both those guidelines and the law’s reflection in OHIP policies, which does not extend clitoral reconstructive surgery to them.

## Discussion

This study revealed how a small sample of women with FGC’s reproductive health care experiences in Toronto were shaped by views about the normal body in laws (Bill C-27 and practice guidelines) and standardized medical coverage (OHIP). Women with FGC in this study wanted clitoral reconstructive surgery but were unable to access it. OB/GYNs in the present sample too were unable to legally provide reinfibulation nor provide clitoral reconstructive surgery covered by medical insurance. Thus, there is a tension between what the law and practice guidelines imply—an adult woman’s ownership of her body—and what the law allows (particularly Bill C-27). Together, the law and related policy shaped participating women with FGC’s feeling of being not “normal” and being excluded in reproductive health care, as well as participating doctors’ ethical dilemmas in treating these patients. The “inconsistencies in the law” which we discuss have also been described in scholarship from the UK on debates about FGC (Dustin, 2010, p. 7) and legislation surrounding FGC (Shahvisi, 2017).

The importance of being accepted as “normal” is not new to the literature. In fact, the practice that women with FGC originally went through to be accepted in natal countries often makes them different in Canada (Jacobson et al., 2018). While other studies have also described the tension of no longer fitting in with natal norms and feeling othered and different in diaspora (Berggren, Bergström, et al., 2006; Berns-McGown, 1999; Jacobson et al., 2018; O’Neill & Pallitto, 2021), this study links this tension to the law and related policy, which juxtaposes women’s rights and freedoms with limits on what they can do with their bodies.

Despite women in this study wanting to undergo clitoral reconstructive surgery, which would make their vulvar appearance closer to that of the uninfibulated vulva without FGC common in the West, it was still not financially accessible to them. Therefore, participants with FGC were frustrated; they were unable to make their vulva “normal,” resembling those of women without FGC. The vulvas of women with FGC are thus left in limbo—not consistent with natal or diasporic conceptualizations of “normal” (Jacobson et al., 2018). This means that women in this study were neither able to instantiate what was “normal” in their natal cultures because reinfibulation is prohibited by Bill C-27, nor what was “normal” in Canada because of their lack of access to clitoral reconstructive surgery. This caused women with FGC who participated in this study distress and contributed to their sense of being unaccepted.

What is conceived of in the West as a “normal” vulva, as perpetuated by Bill C-27, often becomes a new standard to which women with FGC compare themselves. This is not the first study in which participating women with FGC place emphasis on the importance of reconstructive surgery for the purpose of not only improving their sexual health, but also to “just be accepted” and to be “normal” (Jordal et al., 2019). This finding is not consistent with the popular perception that women with FGC will seek out reinfibulation. This finding is also in contrast to other research that discusses some women’s preference to stay infibulated (Johansen, 2019).

In the current study, both participating women with FGC and OB/GYNs compared the limitations on the genital surgeries to which women with FGC have access to similar surgeries afforded to Western individuals. In particular, women with FGC in the present sample compared their lack of access to clitoral reconstructive surgery to gender confirming surgery, which they knew could be covered for trans individuals. This comparison was made to highlight what was in the realm of possibility in terms of OHIP coverage for themselves versus others. However, the women in this study were not necessarily aware of the difficulties and burdens that trans individuals encounter when accessing gender confirming surgery, including their navigation of standardized protocols and mental health assessments meant to help doctors explore alternative diagnoses (MacKinnon et al., 2019; MacKinnon et al., 2020). It would be beneficial for further research to investigate how the difficulties that trans individuals face when accessing covered gender confirming surgery might be similarly reflected in the barriers that women with FGC may face if their reconstructive surgery becomes paid for by OHIP.

The language of “harassment” and not “normal” in description of FGC contributes to anti-FGM discourse, in which the practices, women, and related cultures are depicted as “barbaric” (Jacobson et al., 2021), thus perpetuating the stigmatization of women with FGC. In particular, the use of stigmatizing language in the law to describe FGC does not consider what is “normal” within the varying cultures to which the Canadian government grants citizenship. It is no coincidence then that the same inequities are mirrored in OHIP coverage, which allow particular populations access to covered genital modification (i.e., trans individuals), but do not allow women with FGC coverage for clitoral reconstructive surgery. Therefore, the law sets the standard for the degree of inclusivity and exclusion in related policies. Perhaps the genital surgeries that participants desired but were not funded for by OHIP would allow them to recognize their body as their own, much like gender confirming surgery may do for trans individuals (Walsh & Einstein, 2020). Therefore, it is time to consider covering reconstructive surgery for women with FGC who want to undergo it.

While some studies report reduced vulvar pain and increased sexual pleasure for women who have had reconstructive surgery (Abdulcadir et al., 2015a; Foldès et al., 2012), other work reports that “sexuality-related outcomes” are worse in approximately 22% of women after reconstructive surgery (Berg et al., 2018, p. 278). In addition, one case study reported post-traumatic stress from reconstructive surgery due to infection post-operatively (Abdulcadir et al., 2017). The WHO (2016) has also acknowledged both the potential benefits (e.g., improvement of “chronic clitoral pain” and “dyspareunia”; p. 32) and risks (“damage to neighbouring structures such as the urethra and the clitoral neurovascular bundle”; p. 32) of reconstructive surgery. Thus, the literature is controversial, which underscores the importance of considering the risks as well as the perceived benefits of the procedure.

There has also been progress to better understand the safety/efficacy of the surgery (Abdulcadir et al., 2015b) and the benefits of multidisciplinary counselling for women prior to surgery (De Schrijver et al., 2016). In addition to the need to better understand reconstructive surgery outcomes for women with FGC, there is also a need to better instantiate equity within OHIP policy, which implicitly mirrors inequities perpetuated by the law. Achieving this would involve allowing women with FGC seeking clitoral reconstructive surgery the same coverage as is afforded to other groups seeking genital surgeries. Aside from financial coverage, another element to accessibility was participants’ potential inability to find a surgeon expert in performing clitoral reconstructive surgery. This may indicate a need for more training for health care providers around provision of this surgery, including its risks and benefits, and the importance of counselling for women with respect to the surgery (De Schrijver et al., 2016).

It is important to note that while both clitoral reconstructive surgery and FGCS are legal, both procedures are not usually covered by health insurance and are accessible to only those who have the means to pay (Boddy, 2016, 2020). However, unlike clitoral reconstructive surgery, in some infrequent cases in which there are functional reasons, Western women may be afforded coverage for FGCS in Canada. Part of further exploring inequities when it comes to clitoral reconstructive surgery (especially in the context of the anti-FGM discourse to which women are exposed in the West; Jacobson et al., 2021) includes grappling with the tension in the literature regarding Western social and body norms that may drive women to seek clitoral reconstructive surgery in order to instantiate “normality”—a driving force which may also influence some women seeking FGCS (Johnsdotter, 2020).

Future research in the Ontario context specifically may focus on how OHIP policies surrounding clitoral reconstructive surgery not only reflect but are more concretely shaped by the law. Additionally, there is still a gap for understanding whether the inequities in funding by OHIP also exist in other provinces or countries with different health care system organization. Part of further exploring OHIP policies and the law includes further investigation of outcomes and better definition of the circumstances in which reconstructive surgery may be considered as medically necessary and when it may be considered as cosmetic.

OB/GYNs in this study made a different comparison of genital procedures, particularly, reinfibulation and FGCS. Both were viewed as cosmetic procedures that alter the labia. This is not the first study to question what has been called the “moral double standard” in which reinfibulation is illegal, but FGCS are accepted for consenting adults (Ahmadu & Shweder, 2009; Earp, 2016, p. 105; Johnson-Agbakwu & Manin, 2021, p. 1951; Manderson, 2004). Importantly, recent literature suggests that there is the possibility that women who are infibulated can “live well” (Johnsdotter & Essén, 2021, p. 1944). The idea that women can live well while infibulated is new to the literature and relevant here because it counters the idea in the law that infibulation is *strictly* “mutilation” or something that causes sustained bodily harm. However, in this article, we do not suggest or advocate for the legalization of reinfibulation. We instead wish to draw attention to inequities in the law. These inequities limit an individual’s ability to modify their reproductive body to conform only to the Western conceptualization of “normal.”

Our findings question Canada’s commitment to multiculturalism through the Canadian Multiculturalism Act (announced in 1971 and enacted in 1988; Canadian Multiculturalism Act, 1990; Wayland, 1997) when it comes to diverse bodies. This is not the first call for a greater acceptance of “biologies of diversity” (Einstein, 2012). It is important to consider how what is “written on the body” (Einstein, 2012) becomes political in the face of discriminatory and inequitable discourse and laws, which point to a normalization of women’s bodies (Boddy, 2020; Manderson, 2004). This normalization may also affect women without FGC. It is therefore pertinent to develop a greater acceptance, reflected in the law and policy, of the body shaped by FGC—infibulated or deinfibulated. This includes affording women with FGC the same right to accessible health care and treatment as other Canadians without FGC. It is important to develop this kind of acceptance of diverse bodies in and beyond women with FGC to work toward their inclusion in Canadian society and to ensure the “human-rights and fundamental freedoms” guaranteed to them under the law (Bill C-27, p. 1).

## Conclusion

This study investigated the social relations that shaped women with FGC’s reproductive health care experiences in Toronto. Women with FGC in this study experienced inequities in the genital surgeries that they were unable to access (i.e., clitoral reconstructive surgery), which revealed the disjuncture that the practice of inclusivity is more complicated than the rhetoric of it. This was similarly reflected in participating OB/GYNs’ descriptions of the genital surgeries they could and could not legally perform. We linked up this disjuncture to Bill C-27, which limited what adult women with FGC could do with their bodies. This limitation was reflected in OHIP policies that did not cover women with FGC in this study for clitoral reconstructive surgery, shaping their experience of exclusion in the reproductive health care system. The law also shaped participating doctors’ ethical dilemmas with regard to the genital surgeries that they could not legally provide.

This study highlights the importance of developing more acceptance and celebration of diverse bodies and questions inequities within the law that shape women with FGC’s exclusion in the reproductive health care system and doctors’ ethical dilemmas. Ensuring that women who have undergone FGC have the same claim to their fundamental freedoms will help to work toward the inclusion of those with bodies that are considered to be diverse in Canadian society and toward equity in our reproductive health care system.

## Data Availability

All relevant data are present in direct quotes throughout the article. Transcripts and audio recordings of interviews are not made available to protect the privacy of participants.
